# Report of a Case of Triplets

**Published:** 1855-02

**Authors:** R. A. F. Penrose


### *Report of a Case of Triplets.*

By R. A. F. Penrose, M.D.

The case reported below, is an abstract made from the notes of Mr. C. H. Hall, a member of my practical obstetrical class, and occurred in one of the patients assigned to his charge. It is given merely to increase the statistics on the subject, and because the mechanism of labor was carefully watched by Mr. Hall.

"I was called, Dec. 28th, 1854, to Mrs. W——, supposed to be in labor, this being her second confinement. Found that she had been suffering, more or less, with pains since the midnight previous. My attention was called at once to what she termed "a great swelling in her side," and on examining her abdomen, an unusual prominence was found extending from the hypogastric into the right lumbar region, apparently produced by the position of the child in utero. The os uteri was found slightly dilated, soft and moist. From this time, 11 o'clock, A. M., the labor progressed very slowly. By four o'clock, the head had somewhat descended, and the occiput was found in front and to the left, and by six, the child was born. The cord was very short. On applying the hand upon the abdomen, to ensure contraction of the uterus, it was found to be of very unusual size, and suspecting another child, I made an examination, and found a head presenting *exactly in the same position* as the first. The pains being very feeble, the delivery of the head of the second child was very tedious, the head remaining almost three quarters of an hour half protruded through the vulva. Some ergot being administered, the uterine contractions became more powerful, and the head was expelled. And now a hard, round tumor was felt distending the perineum, and as the shoulders of the second child were born, the head of a third slipped out, which had descended, firmly pressed against the anterior and upper portion of the thorax of the second child, and having the same position as the heads of the two preceding children, viz.: the occiput to the front. The two children thus born simultaneously, exhibiting no signs of life, every effort made to resuscitate them proving unavailing. Particular attention was paid to the uterus, in order to secure proper contraction, and more ergot was given to promote the same object, the patient being very much exhausted. The placenta was delivered, and on examination, it was found that there was a common placental mass for the three children. This mass was more than double the size of an ordinary placenta, was of oblong shape, having three cords, and the remains of three bags of membranes. Upon injecting fluid into the different cords, it was found that the fluid injected into the vessels of one cord could not be made to pass out by the vessels of either of the others, but returned by the vessels of the same cord.

The children were all small. The mother did well.

